# 

**Published:** 1850-08

**Authors:** 


### CONTENTS

### OF NO. II.

### ORIGINAL COMMUNICATIONS.

### ESSAYS AND CASES.

Art. I.—Foreign Correspondence of the Western Journal of Medicine and Surgery, - - - - - 93
Art. II.—Brief Notes on Cholera in Louisville in 1850. By Theodore S. Bell, M. D., of Louisville, Ky. 99

### SELECTIONS FROM AMERICAN AND FOREIGN JOURNALS.

On the Dislocation of the Lower Jaw, - - - 105
On the Treatment of Ague by a Single Dose of Quinine, 108
On Mucous or Catarrhal Pneumonia in Children, - - 108
Clinical Convictions respecting Ascites, expressed in the form of Propositions, - - - - - - 110
On Phlebitis, - - - - - - - - 113
On the Hydrostatic Test, - - - - - 113
On the Use and Advantages of Opium in the Practice of Obstetricy, - - - - - - - 114
Report of a Case of Premature Labor, Artificially Induced, with Successful Results, at the Seventh Month of Gestation, on Account of Contracted Pelvis, - - 121
On the Statistics of the Mortality from Fractures of the Head, - 128
Insanity from the Use of Chloroform during Parturition, - 130
Case of Gunshot Wound, and Subsequent Extraction of a Bullet from the Bladder, - - - - - 131
Therapeutical Effects of Turpentine, - - - - 132
Condition of the Ovaries and Uterus, observed in a young woman assassinated shortly after Menstruation, - - 139
Remarks on Vermifuges, - - - - - - 141

Case of Spontaneous Expulsion of the Child, - - - 145
Treatment of Dysmenorrhea by Quinine and Prussiate of Iron, 148
Medicine in Spain, - - - - - - 149
Dr. Webster and his Spiritual Adviser, - - - 150
Proceedings of the American Medical Association, at the Third Annual Meeting, held at Cincinnati, May, 1850, - 151

ORIGINAL INTELLIGENCE.

Changes in Medical Schools, - - - - - 178
The New York Medical School, - - - - 180
The Ohio Medical School, - - - - - 181
Obituary, - - - - - - - 181
The Western Lancet and Hospital Reporter, - - 181
Dr. Latta's Pamphlet on Homœopathy, - - - 182
Chemical Instruction, - - - - - 182
Medical Schools, - - - - - - 183
Apology to the readers of the Journal of Medicine, - 184
Foreign Correspondence, - - - - - 184

